# Human adipose tissue accumulation is associated with pro-inflammatory changes in subcutaneous rather than visceral adipose tissue

**DOI:** 10.1038/nutd.2017.15

**Published:** 2017-04-10

**Authors:** I Kralova Lesna, S Cejkova, A Kralova, J Fronek, M Petras, A Sekerkova, F Thieme, L Janousek, R Poledne

**Affiliations:** 1 Laboratory for Atherosclerosis Research, Institute for Clinical and Experimental Medicine, Centre for Experimental Medicine, Prague, Czech Republic; 2 Transplant Surgery Department, Institute for Clinical and Experimental Medicine, Prague, Czech Republic; 3 2nd Faculty of Medicine, Charles University, Prague, Czech Republic; 4 Department of Clinical and Transplant Immunology, Institute for Clinical and Experimental Medicine, Prague, Czech Republic

## Abstract

The importance of the involvement of adipose tissue macrophage subpopulations in obesity-related disorders is well known from different animal models, but human data are scarcer. Subcutaneous (*n*=44) and visceral (*n*=52) adipose tissues of healthy living kidney donors were obtained during living donor nephrectomy. Stromal vascular fractions were isolated and analysed by flow cytometry using CD14, CD16, CD36 and CD163 antibodies. Total macrophage numbers in subcutaneous adipose tissue increased (*P*=0.02) with body mass index (BMI), with a similar increase seen in the proportion of phagocytic CD14+CD16+CD36^high^ macrophages (*P*<0.01). On the other hand, there was an inverse correlation between anti-inflammatory CD14+CD16−CD163+ macrophages (*P*<0.05) and BMI. These correlations disappeared after excluding obese subjects (BMI ⩾30 kg m^−2^) from the analysis. Interestingly, none of these subpopulations were significantly related to BMI in visceral adipose tissue. Obesity *per se* is associated with distinct, highly phagocytic macrophage accumulation in human subcutaneous adipose tissue.

## Introduction

Adipose tissue is composed of distinct cell types in addition to adipocytes. Adipocyte expansion is associated with immune cell recruitment of obese adipose tissue both in experimental models and in man^1, 2^ and a close interplay between immune cells and adipocytes has been proven.^3, 4^ New findings highlight the principal role of macrophages in obesity-driven changes of adipose tissue,^5^ including (but not limited to) hypoxia, altered secretion profiles and inflammatory changes (reviewed in Exley *et al.*^6^). These inflammatory changes include quantitative and qualitative changes of macrophage activation states^7^ and participate in pathophysiological changes in adipose tissue.^8, 9^ A significant number of ATMs are also present in lean, metabolically normal subjects,^1^ which suggests that not all ATMs are pro-inflammatory and negatively affect metabolic function. The interplay of pro-inflammatory and anti-inflammatory macrophages might influence the ultimate effect of adipose tissue cumulating triglycerides in obesity.

The aim of our study was to identify obesity-related changes of proportions of these two phenotypes in adipose tissue macrophage subpopulations in healthy subjects.

## Materials and methods

Subcutaneous (SCAT) and visceral (VAT) (outside Gerota's fascia) were obtained from living kidney donors intra-operatively during hand-assisted retroperitoneoscopic living donor nephrectomy. All individuals were of Caucasian origin. The design of the study was approved by the Institute's Ethics Committee. All participants were fully informed about the study and signed informed consent forms. Samples of SCAT and VAT adipose tissues were collected during hand-assisted laparoscopic nephrectomy, cooled, and immediately transferred to the laboratory. The stromal vascular fraction was separated using a procedure according to Zuk *et al.*^10^ with a minor modification.^11^ Briefly, after removing visible blood vessels and connective tissues, each tissue sample was dissected into small pieces, exposed to collagenase and then repeatedly filtered and purified. Stromal vascular fraction was separated and analysed the same day using a CyAn flow cytometry analyser (Beckman Coulter, Brea, CA, USA). Different monoclonal antibodies and fluorochromes (CD14—Phycoerythrin-Cyanine 7 (PC7), CD16- Phycoerythrin-Texas Red-X, ECD, CD 36—Fluorescein isothiocyanate, FITC, CD 163 Phycoerythrin, PE/clone RM3/1 CD 163) were used to identify different subsets of monocytes/macrophages (Supplementary Figure 1). Flow cytometry data were analysed using Kaluza software (Beckman Coulter).

Total cholesterol and triglycerides were determined in fasting blood (minimally 12 h) samples obtained immediately before operation (before anaesthesia) using an enzymatic method (Hoffman-LaRoche, Basel, Switzerland). High-density lipoprotein cholesterol concentrations were analysed (Cobas Mira Plus, Roche, Switzerland) after precipitation of apoprotein B-containing particles using the phosphotungstate method.

Data are presented as means with s.d.'s for continuous variables and percentages with s.d.'s for categorical variables. Inter-group comparisons of continuous variables were performed using the unpaired *t*-test. Linear regression was used to model the relation of the proportion of macrophages to BMI. In all tests, *P*-values less than 0.05 were considered statistically significant.

## Results

The total number of living kidney donors was 52 (19 men and 33 women), their anthropometric and biochemical characteristics are shown in Table 1. The total number of adipose tissue samples analysed was 44 for SCAT and 52 for VAT. Because of the recently more flexible criteria for living kidney donation, the prevalence of mild hypertension was around 20%, overweight 36% and obesity 13%. The prevalence of increased LDL concentrations was 8%, decreased HDL concentrations (lower than 1.0 mmol l^−1^ in men and 1.3 mmol l^−1^ in women) were present in 58% and hypertriglyceridaemia (more than 1.6 mmol l^−1^) in 26% of subjects. Five of the total of 52 subjects used statins, while two subjects were taking antihypertensive drugs and one subject used antidepressants.

The total number of macrophages per gram did not differ significantly between SCAT and VAT (10 900±12 250 vs 13 200±10 350, respectively). When dividing stromal vascular fraction macrophages by CD16 positivity, the higher proportion of CD16 positive macrophages in VAT compared to SCAT was significant (*P*<0.05, Table 2). Based on data from the literature^1, 7, 8, 12^ and our recent results,^11, 13^ we defined pro-inflammatory, highly phagocytic macrophages in adipose tissue as CD14+CD16+CD36^high^. In contrast, macrophages with no expression of CD16 but with CD163 positivity were defined as anti-inflammatory macrophages.^14, 15^ At the same time, we are well aware that this classification could oversimplify *in vivo* conditions whereby the full phenotypic spectrum of transient phenotypes between M1 and M2 macrophages exists.

A slightly lower mean proportion of highly phagocytic CD14+CD16+CD36^high^ macrophages was found in SCAT when compared to VAT (*P*<0.02, Table 1), whereas the proportion of anti-inflammatory macrophages (defined as CD14+CD16−CD163+) did not differ significantly. The remaining (transient) macrophage subpopulations included 15–20% of total macrophages.

**Figure 1:**
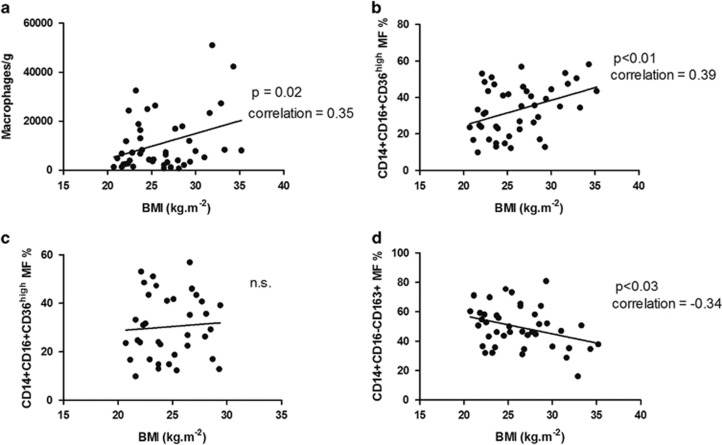
The relationship between macrophage subpopulations (MF) and BMI in subcutaneous adipose tissue. The relationships of macrophages and BMI in SCAT. The relationship between total macrophages and BMI (**a**); the relationship between pro-inflammatory macrophages CD14+CD16+CD36^high^ and BMI (**b**); the same relationship in non-obese subjects (**c**); the relationship between anti-inflammatory CD14+CD16−CD163+ macrophages and BMI (**d**).

There was a significant association (*P*=0.02) of BMI and the total number of macrophages isolated from stromal vascular fraction of SCAT (Figure 1a). The proportion of pro-inflammatory CD14+CD16+CD36^high^ macrophages correlated positively with BMI of the all subjects included (*P*<0.01, Figure 1b). We speculated that this significant correlation might be due to seven obese individuals. When considering only the non-obese (BMI⩽30 kg m^−2^), the positive correlation between BMI and pro-inflammatory macrophages disappeared (Figure 1c). On the other hand, there was a borderline significant relation of BMI to anti-inflammatory CD14+CD16−CD163+ macrophages (*P*<0.03, Figure 1d) but this correlation also disappeared after excluding subjects with obesity (data not shown). A detailed analysis of macrophage subsets comparison in lean, overweight and obese subjects is available in Table 1 and Supplementary Material.

All of the above measures were analysed also for VAT documenting a similar pattern; however, without reaching statistical significance (Table 2 and Supplementary Material).

The association of waist circumference with macrophage subpopulations was also analysed in both adipose tissues (Table 2 and Supplementary Material) and showed a pattern similar to that of BMI.

We also performed an analysis for all other macrophage subpopulations with proportions higher than 5%, but none of these were related to BMI or waist circumference.

## Discussion

The living kidney donor nephrectomies provided us with a unique opportunity to compare the importance of different types of human adipose tissue in healthy subjects. Our main finding was the prominent role of macrophages in SCAT in obese individuals. Likewise, we observed a specific proportion of highly phagocytic macrophages (CD14+CD16+CD36^high^) positively associated with BMI. On the contrary, there was an inverse correlation between anti-inflammatory macrophages (CD14+CD16−CD163+, corresponding to M2 macrophages) and BMI. Interestingly, no such relation was found in VAT.

When distinguishing the ATM subpopulations by CD16 only, the proportions of subpopulations were not significantly associated with increased BMI. This is in agreement with data reported in a study of a small group of extremely obese volunteers,^7^ where dietary intervention failed to change the proportion of ATM in SCAT when distinguished only by the CD16 marker. However, the addition of other markers (CD36 and CD163) revealed a significant correlation of pro-inflammatory and anti-inflammatory macrophages to BMI. This clearly documents the importance of these markers for the measurement of adipose tissue inflammation estimated as the presence of pro-inflammatory macrophages. Of note, when excluding all individuals with obesity (BMI more than 30 kg m^−2^), the correlation of pro-inflammatory macrophages with BMI (Figure 1c) as well as the negative correlation of anti-inflammatory macrophages (data not shown) disappeared. It is of interest that the arbitrary borderline of obesity corresponded with our data, documenting the transition of adipose tissue toward inflammation in subjects with obesity. Although, in our study, total macrophage number per gram also increased with BMI, we believe that the analysis of balance of the pro- and anti-inflammatory macrophage subpopulations is more informative.

As the association of obesity to pro-inflammatory changes has been repeatedly proven, it is reasonable to assume that the macrophage subpopulations correlating with obesity correspond to the proportions of M1 and M2 phenotypes. However, macrophage cells are highly adaptive to their tissue environment and adipose tissue macrophage subpopulation phenotypes may be tissue-specific. Therefore, our definition of macrophage subpopulations could be influenced by macrophage adaptation to the lipid-rich environments in adipose tissue. As CD36 is a fatty acid transporter, the increase of CD36^high^ macrophages could reflect an increase of the buffer capacity of these cells within an obese adipose tissue environment with high intercellular concentrations of free fatty acids (as recently suggested^16^). Further, it has been shown that CD36 expression in macrophages can increase under activation in a ‘metabolically unhealthy' environment, that is, high concentrations of glucose, insulin and palmitate^8^ contributing to diet-induced insulin resistance.^17^

It is widely accepted that the impact of visceral fat on metabolic and cardiovascular disorders is more important than that of subcutaneous fat, which is why an additional marker of central obesity, that is, waist circumference, is frequently used. Surprisingly, our data show more significant associations of pro- and anti-inflammatory macrophages with BMI and waist circumference in SCAT than in VAT. However, other authors have also found the higher association of SCAT macrophage infiltration^18, 19^ with markers of insulin resistance when comparing SCAT and VAT. Very recent data from Moreno–Indias^20^ also demonstrated that pro-inflammatory changes in SCAT may play a more important role compared to VAT as the metabolic syndrome begins to develop. Based on our results, we suggest that the obesity-related pro-inflammatory changes in metabolically healthy subjects dominantly take place in subcutaneous adipose tissue.

## Figures and Tables

**Table 1:** Characteristics of living kidney donors group (*N*=52)

	*Mean±s.d.*
Age (years)	46.0±10.6
BMI (kg m^−2^)	25.9±3.6
Waist circumference (cm)	90±14
Cholesterol (mmol l^−1^)	4.35±0.88
HDL-cholesterol (mmol l^−1^)	1.19±0.36
LDL-cholesterol (mmol l^−1^)	2.53±0.78

Data are expressed as mean±s.d. Data of waist circumference were available for 39 subjects only.

**Table 2:** Characteristics of macrophages isolated from SCAT and VAT

*Macrophages*	*SCAT (*n*=44)*	*VAT (*n*=52)*	*Significance SCAT vs VAT*
Macrophage CD14+ number per g	10 900±12 250	13 200±10 350	n.s.
CD14+CD16+ (%)	48.9±14.3	53.4±13.3	*P*<0.05
CD 14+CD16+CD36^high^ (%)	32.7±13.8	39.4±13.4%	*P*<0.02
CD14+CD16−CD163+ (%)	49.7±14.1	45.2±13.6	n.s.

Abbreviation: N.s., non-significant.

Macrophages isolated from SCAT and VAT, their proportions and comparison in subgroups. Results are expressed as a mean of the proportion±s.d. The significance was determined using paired Student's *t*-tests. Due to technical problems, quantification of macrophages per g was performed in only 42 (out of 44 samples) in SCAT and in 49 (out of 52) in VAT.
