# Supplementary material for: Human adipose tissue accumulation is associated with pro-inflammatory changes in subcutaneous rather than visceral adipose tissue
Source: Nutr Diabetes. 2017 Apr 10;7(4):e264–. doi: 10.1038/nutd.2017.15 (PMC5436095; doi:10.1038/nutd.2017.15)
Supplement: Supplementary Information [file nutd201715x1.docx]

Supplementary Information, Table 1: Characteristics of macrophages isolated from subcutaneous (SCAT) and visceral (VAT) adipose tissues.

|  | A |  | B |  |
| --- | --- | --- | --- | --- |
| Macrophages | SCAT (n=44) | p | VAT (n=52) | p |
| Macrophage number *per* g | 10 900±12 250 |  | 13 200±10 350 |  |
| CD14+CD16+ (%) | 48.9±14.3 |  | 53.4±13.3 |  |
| CD14+CD16+36^high^ (%) | 32.7±13.8 |  | 39.4±13.4% |  |
| - CD14+CD16+CD36^high^ (%) in lean subjects | 29.3±13.8 | a- _**_ | 35.5±13.2% | n.s. |
| - CD14+CD16+CD36^high^ (%) in overweight subjects | 32.3±12.9 | b- _**_ | 43.4±13.1% | n.s. |
| - CD14+CD16+CD36^high^ (%) in obese subjects | 46.1±9.0 |  | 44.5±10.7% | n.s. |
| - CD14+CD16+CD36^high^ /g in lean subjects | 3 433±4 648 | a- _**_ | 6 654±9283 | n.s. |
| - CD14+CD16+CD36^high^ /g in overweight subjects | 2 619±1 901 | b- _***_ | 5753±4 919 | n.s. |
| - CD14+CD16+CD36^high^ /g in obese subjects | 11 880 ±9750 |  | 5 400 ±3 690 | n.s. |
| CD14+CD16-CD163+ (%) | 49.7±14.1 |  | 45.2±13.6 | n.s. |
| - CD14+CD16-CD163+(%) in lean subjects | 51.2±13.6 | a- _*_ | 49.7±13.6 | n.s. |
| - CD14+CD16+CD163+ (%) in overweight subjects | 52.4±13.8 | b- _*_ | 40.9±10.9% | n.s. |
| - CD14+CD16-CD163+ (%) in obese subjects | 37.2±11.7 |  | 40.0±18.2% | n.s. |

Table 1: Macrophages isolated from SCAT and VAT, their proportions and comparison in subgroups. Results are expressed as a mean of the proportion ±SD. p: a – comparison of lean (n=28, BMI≥25 kg.m^-2^) to obese (n=7, BMI>30 kg.m^-2^), b – comparison of non-obese (n=45, BMI ≤30 kg.m^-2^) to obese. The significance was determined using the unpaired (p) parametric t-Student’s t-tests. Statistical significance levels are given as * p<0.05, ** p<0.01, *** p<0.0001, n.s. = non-significant. Due to technical problems, quantification of macrophages *per* *g* was performed only in 42 (out of 44 samples) in SCAT and in 49 (out of 52) in VAT.

Supplementary Information, Table 2: Relationships of macrophage number and subpopulations proportions to BMI and waist circumference

|  | **BMI** |  | **Waist circumference** |  |
| --- | --- | --- | --- | --- |
|  | p | r | p | r |
| **SCAT** |  |  |  |  |
| Total macrophages *per g* | **=0.02** | **0.35** | **<0.015** | **0.42** |
| CD14+CD16+CD36^high^ (%) | **<0.01** | **0.39** | **=0.01** | **0.42** |
| CD14+CD16-CD163+ (%) | **<0.03** | **-0.34** | 0.12 | -0.26 |
| **VAT** |  |  |  |  |
| Total macrophages *per g* | 0.96 | 0.00 | 0.65 | 0.08 |
| CD14+CD16+CD36^high^ (%) | 0.15 | 0.20 | 0.10 | 0.28 |
| CD14+CD16-CD163+ (%) | 0.27 | -0.16 | 0.19 | -0.22 |

Table 2. Relationship of number and selected subpopulations of macrophages isolated from SCAT (n=44) and VAT (n=52) to BMI and waist circumference. Results are expressed as a statistical value of the relationship (p) and correlation coefficient (r). Waist circumference to macrophage subpopulations data were available only for 39 of VAT samples and only 36 of the SCAT samples analyzed. Due to technical problems, quantification of macrophages *per* *g* was performed only in 42 (out of 44 samples) in SCAT and in 49 (out of 52) in VAT.

Supplementary Information, Figure 1: **Example of SVF flow cytometric analysis.**

**
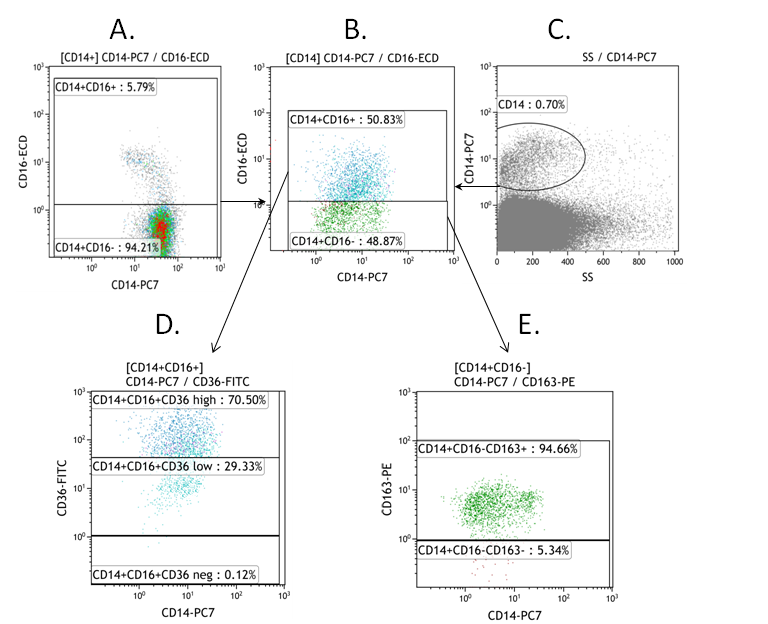
**

Figure 1: **Example of SVF flow cytometric analysis.**

**Legend:** (A) CD16 positive monocytes were first identified and delineated in the blood sample (left, CD16 positive macrophages in the upper part). The settings were fixed and subsequently used for SVF analysis (B). Total macrophages in SVF were identified by positivity for CD14 (C) and, based on the CD16 marker, two subpopulations were distinguished (B, CD16-positive macrophages in the upper part). The CD16+ subpopulation was divided according to the CD36 marker (D), with the highly positive subpopulation at the top and the low-positive in the middle (based on blood macrophage analyses). (E) CD163 expression was determined within the CD16 negative subpopulation and divided (CD163 positive macrophages in the upper part).This scheme is partly simplified, as a few minor fractions (already measured) are not mentioned.
