# Supplementary material for: Human adipose tissue accumulation is associated with pro-inflammatory changes in subcutaneous rather than visceral adipose tissue
Source: Nutr Diabetes. 2017 Apr 10;7(4):e264–. doi: 10.1038/nutd.2017.15 (PMC5436095; doi:10.1038/nutd.2017.15)
Supplement: Supplementary Information [file nutd201715x2.pdf]

blood vessels and connective tissues, each tissue sample was dissected into small pieces, exposed to collagenase and then repeatedly filtered and purified. SVF was separated and analysed the same day using a CyAn flow cytometry analyser (Beckman Coulter, Brea, CA, USA). Different monoclonal antibodies and fluorochromes [CD14 – Phycoerythrin-Cyanine 7 (PC7), CD16- Phycoerythrin-Texas Red-X, ECD, CD 36 – Fluorescein isothiocyanate, FITC, CD 163 Phycoerythrin, PE/clone RM3/1 CD 163-] were used to identify different subsets of monocytes/macrophages (see Figure S1). Flow cytometry data were analysed using Kaluza software (Beckman Coulter, Brea, CA, USA).

Total cholesterol and triglycerides were determined in fasting blood (minimally 12 hrs) samples obtained immediately before operation (prior to anaesthesia) using an enzymatic method (Hoffman-LaRoche, Switzerland). High-density lipoprotein cholesterol concentrations were analysed (Cobas Mira Plus, Roche, Switzerland) after precipitation of apoprotein B-containing particles using the phosphotungstate method.

Data are presented as means with standard deviations for continuous variables and percentages with standard deviations for categorical variables. Inter-group comparisons of continuous variables were performed using the unpaired t-test. Linear regression was used to model the relation of the proportion of macrophages to BMI. In all tests, p values less than 0.05 were considered statistically significant.

3. Results

The total number of living kidney donors was 52 (19 men and 33 women), their anthropometric and biochemical characteristics are shown in Table 1. The total number of adipose tissue samples analysed was 44 for SCAT and 52 for VAT. Due to the recently more flexible criteria for living kidney donation, the prevalence of mild hypertension was around 20%, overweight 36%, and obesity 13%. The prevalence of increased LDL concentrations
